# A Review on the Main Phytoconstituents, Traditional Uses, Inventions, and Patent Literature of Gum Arabic Emphasizing *Acacia seyal*

**DOI:** 10.3390/molecules27041171

**Published:** 2022-02-09

**Authors:** Mohamed A. Ashour, Waseem Fatima, Mohd. Imran, Mohammed M. Ghoneim, Sultan Alshehri, Faiyaz Shakeel

**Affiliations:** 1 Department of Phytochemistry and Natural Products, Faculty of Pharmacy, Northern Border University, Rafha 91911, Saudi Arabia; ashourmohamed08@yahoo.com; 2 Department of Pharmacognosy, Faculty of Pharmacy, Al-Azhar University, Nasr City 11884, Egypt; 3 Department of Clinical Nutrition, Northern Border University, Arar 91431, Saudi Arabia; fatimawaseem1512@gmail.com; 4 Department of Pharmaceutical Chemistry, Faculty of Pharmacy, Northern Border University, Rafha 91911, Saudi Arabia; 5 Department of Pharmacy Practice, College of Pharmacy, AlMaarefa University, Ad Diriyah 13713, Saudi Arabia; mghoneim@mcst.edu.sa; 6 Department of Pharmaceutics, College of Pharmacy, King Saud University, Riyadh 11451, Saudi Arabia; salshehri1@ksu.edu.sa

**Keywords:** gum Arabic, *Acacia seyal*, Arabic gum, invention, patent

## Abstract

*Acacia seyal* is an important source of gum Arabic. The availability, traditional, medicinal, pharmaceutical, nutritional, and cosmetic applications of gum acacia have pronounced its high economic value and attracted global attention. In addition to summarizing the inventions/patents applications related to gum *A. seyal*, the present review highlights recent updates regarding its phytoconstituents. Traditional, cosmetic, pharmaceutical, and medicinal uses with the possible mechanism of actions have been also reviewed. The patent search revealed the identification of 30 patents/patent applications of *A. seyal*. The first patent related to *A. seyal* was published in 1892, which was related to its use in the prophylaxis/treatment of kidney and bladder affections. The use of *A. seyal* to treat cancer and osteoporosis has also been patented. Some inventions provided compositions and formulations containing *A. seyal* or its ingredients for pharmaceutical and medical applications. The inventions related to agricultural applications, food industry, cosmetics, quality control of gum Arabic, and isolation of some chemical constituents (L-rhamnose and arabinose) from *A. seyal* have also been summarized. The identification of only 30 patents/patent applications from 1892 to 15 November 2021 indicates a steadily growing interest and encourages developing more inventions related to *A. seyal*. The authors recommend exploring these opportunities for the benefit of society.

## 1. Introduction

The genus *Acacia* (also known as wattles) is a large genus formed mainly of shrubs and trees that belong to the subfamily Mimosoideae and the pea family (Fabaceae). Plant species of this genus grow natively in the tropical and subtropical regions of the world, including Africa, Australia, middle America, the Middle East, and south Asia. The genus name “*Acacia*” was introduced by Philip Miller in 1754 [1] and is derived from the Greek name (ἀκακία) “akakia”, a term used by Dioscorides (40–90 AC) for a prepared extract from leaves and pods of *Acacia nilotica* “*Vachellia nilotica*”. The genus *Acacia* formerly contained 1540 species as recognized in 2011. However, these plant species were later divided into five clades (genera) after a long controversial debate [2,3,4,5]. The clades are varied in species count and habitat: Acaciella Britton & Rose (15 species) and Mariosousa Seigler & Ebinger (13 species) are confined to the Americas. Vachellia Wight & Arn. (163) and Senegalia Raf. (194 species) are pantropical (mainly in Africa and India). The largest clade, corresponding to *Acacia* following the Vienna Congress, comprises 1021 species, almost all of which are Australian [6]. However, there are a great number of botanists who conserve the old nomenclature and disagree with this recent classification [7].

*Acacia seyal* Del. (homotypic synonym: *Vachellia seyal* Del.; another synonym: *Acacia stenocarpa* Hochst.; English name: Whistling thorn; Arabic Name: Taleh or Talha) [8] well-known species belongs to the genus *Acacia* (or *Vachellia*), Family *Fabaceae*. Besides the ecological, social, and economic importance of *Acacia* species. *A. seyal* is a well-known traditional medicinal plant that has a wide range of medicinal applications related to its different phytoconstituents from organized parts, e.g., fruits, barks, stem, and roots, and unorganized parts, e.g., gum acacia, which is called “taleh or talha gum” [6].

The bark of *A. seyal* can be easily recognized where *A. seyal* var. *seyal* has thin red-brown bark, while the bark of *A. seyal* var. *fistula* is smooth and whitish. Both varieties have long, slender, and white thorns that occur in pairs; the thorns of *A. seyal* var. *fistula* are sometimes swollen at the base by ant galls. The inflorescence of *A. seyal* is almost yellow, pedunculate with a globose head. Pods are 7–20 cm long, thin, and slightly curved [9].

Some reviews on *A. seyal* and *A. senegal* have been documented, which have been cited at the appropriate places of this manuscript. This review has been written to provide recent updates regarding the phytoconstituents, traditional, cosmetic, pharmaceutical, and medicinal uses of *A. seyal* with the possible mechanism of actions along with an insight of the inventions/patents applications related to gum *A. seyal*.

## 2. Main Phytoconstituents

**Figure 1:**
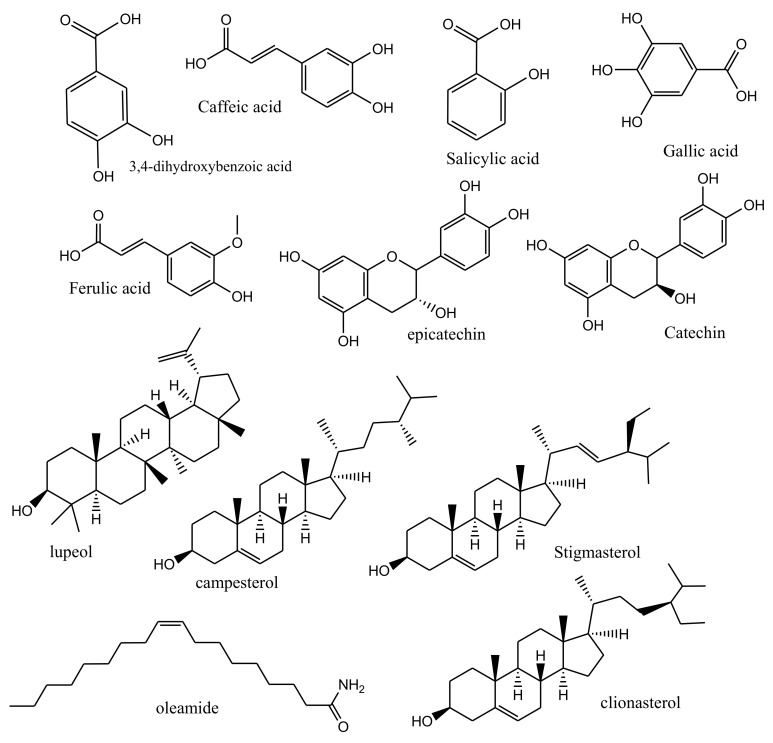
The main phytoconstituents of *A. seyal*.

The chemical composition, including the main phytoconstituents of *A. seyal* (Figure 1), has been established and previously reported, and it can change with its geographical source, age of the trees, weather, and soil conditions [10,11]. Leaves, flowers, and pods of *A. seyal* contain reasonable amounts of phytochemicals, including proteins, saponins, phenolics, flavonoids, anthocyanins, and carbohydrates [12]. Although alkaloids and anthraquinones were not detected in the bark extract of the plant according to Suleiman & Brima 2021 [13]. In other studies, the stem bark has been reported to contain flavonoids, saponins, terpenoids, steroids, alkaloids, phenols, coumarin, and tannins [14,15]. The phenolic acids “gallic acid, salicylic acid, p-coumaric acid, caffeic acid, 3,4 dihydroxy benzoic acid, and ferulic acid” were detected in *A. seyal* leaves [16,17,18]. The stem bark of *A. seyal* (Djibouti type) was reported to contain catechin, epicatechin, lupeol, campesterol, stigmasterol, clionasterol, and oleamide [19], whereas the complex of polysaccharides and calcium, magnesium, potassium salts, protein, gallic, ellagic, and chlorogenic acids were reported as phytoconstituents of *A. seyal* gum [20].

According to Eltayeb et al. 2017 [21], the Sudanese *A. seyal* stem and stem wood contain tannins, terpenoids, cardiac glycosides, reducing sugars, flavonoids, alkaloids, steroids. The stem barks extract shows only positive results for tannins, terpenoids, cardiac glycosides, and reducing sugars, while all test materials are free from saponins. The dry distillates of the stem materials of *A. seyal* (known in Sudan as Dokhan) are used as fumigants for their cosmetic, aromatic, and medicinal values. The GC-MS analysis of this dry distillate revealed the presence of more than 130 volatile constituents, while the major vol. constituents were solerone, furfural, catechol, syringol, allo-inositol, mequinol, furfuralcohol, 3-methyl-1,2-cyclopentanedione, phenol, homovanillyl alcohol, 1,3-dimethyl-5-methoxypyrazol, and 1,2-anhydro-3,4,5,6-alloinositol. [21].

**Figure 2:**
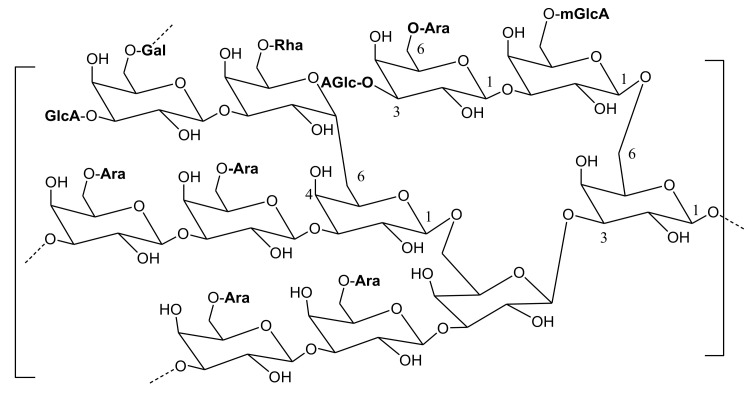
Part of the fundamental chain of gum Arabic shows 1-3-linked β-D-galactopyranosyl residues and its main branches. (Gal) β-D-galactopyranose, (Ara) α-l-arabinofuranose, (Rha) α-l-rhamnopyranose, (GlcA) β-D-glucuronic acid, and (mGlcA) 4-O-methyl-β-D-glucuronic acid.

**Figure 3:**
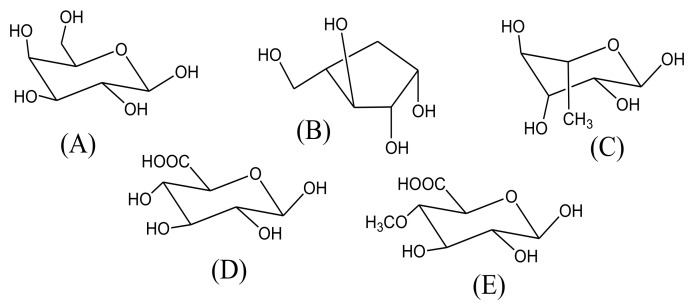
The main monosaccharide residues in gum Arabic: (A) β-D-galactopyranose, (B) α-l-arabinofuranose, (C) α-l-rhamnopyranose, (D) β-D-glucuronic acid, and (E) 4-O-methyl-β-D-glucuronic acid.

Gum Arabic (GA) or acacia gum is dried gummy exudate (mainly shaped in tears, spherical, or subspherical forms) obtained pathologically, mainly by incision, from the stems and stem branches of acacia trees, especially *A. senegal* and *A. seyal*, family Fabaceae. *A. senegal* gum is called “hashab gum” and has a milky white appearance and is hard; while *A. seyal* gum is known as “Talha gum”, which has mainly amber yellow color and is friable [22]. GA is an arabinogalactan-protein complex (known as arabin) which is composed mainly of calcium, magnesium, and potassium salts of Arabic acid. Arabic acid is composed mainly of 1-3-linked β-D-galactopyranosyl units with branches that consist of two to five β-D-galactopyranosyl residues linked together through 1,3-ether linkages and attached to the fundamental β-D-galactopyranosyl chain (Figure 2) through 1,6-linkages. Both fundamental and branches contain additional α- l -arabinofuranosyl and α-l-rhamnopyranosyl units and terminated with β-D-glucopyranosyl and 4-O-methyl-β-D-glucopyranosyl residues (Figure 3).

Compared with *A. senegal* gum, *A. seyal* gum is more compact and friable, less charged, less hydrolyzable by enzymes, less surface-active, more unstable in solution, richer in minerals and polyphenols, and less rich in proteins [23]. In a study reported by Karamalla 1999 [24], GA contains about 10.75% as an average moisture content, which determines the hardness of the gum and average ash content as 3.27% for *A. senegal* var. *senegal* samples, while the average moisture and ash content of *A. seyal* gum was reported to be 14.41% and 3.5%, respectively [25]. The protein content is responsible for the emulsification properties of GA. For good-quality GA, the European specifications and the United States pharmacopeia define that at least 3% of GA should be protein content [11]. However, the percentage of GA proteins is varied according to the geographical source, the constitution of soil, time of collection, and the plant species; for example, the protein contents in GA from Nigerian *A. senegal* contain approximately double the content found in Nigerian *A. seyal* gum, which could explain the instability of the oil in water emulsification properties of *A. seyal* gum [11,26]. *A. senegal* gum contains high amounts of hydroxyproline, serine, leucine, threonine, histidine, and aspartic amino acids compared with lower amino acid contents present in *A. seyal* gum [27]. GA is acidic; its pH is 4.66, as described by Karamalla 1999. [24] The average optical rotation of hashab gum (*A. senegal* gum) is −30°, while the [α]D values of talha gum (*A. seyal* gum) are ranged between +45° to +54° [28].

Although polysaccharides macromolecules are mainly sparingly soluble in water, GA is soluble easily in hot and cold water, forming aqueous concentrated solutions of up to 50% concentration. Like most polysaccharides, GA is insoluble in non-polar organic solvent and oils, but it can be soluble in aqueous ethanol solutions up to 60% ethanol concentration [29]. The mineral types and concentrations in gum Arabic attract important attention as they are responsible for the polarity of the arabinogalactan protein complex and, in turn, have an impact on the solubility, hydration compactness, and stability of the colloidal solution of the gum [11].

Gum talha (Sudanese type) is mainly formed of rhamnose (3–4%) and arabinose (41–45%) in addition to nitrogen contents (0.147–0.175%) and protein (0.97–1.15%). Gum talha has [α]D values ranging between +45° and +54° [28]. However, *A. seyal* gum could be fractionated into three fractions using size exclusion chromatography (SEC) and hydrophobic interaction chromatography (HIC), which were designated as arabinogalactan (AG), arabinogalactan-protein (AGP), and glycoprotein (GP) [30,31]. Li et al. (2020) [25] provided another method for commercial fractionation of *A. seyal* gum using subsequent concentrations of ethanol in distilled water (60% and 80%) to obtain a gum precipitate AY60 and AY80, respectively. In addition to the dried supernatant (AYS), Li et al. (2020) [25] provided analytical data regarding *A. seyal* gum and its these fractions (Table 1).

Further experiments confirmed that the AY60 backbone is composed of 1,3-linked galactopyransyl residues substituted at O-4 and O-6 positions, while the substitutions were 3-1α arabinofuranosyl (~2.25%) or 4-1β glucuronopyranosyl (~14.4%) and terminated by arabinofuranosyl and occasionally by rhamnopyranosyl or glucuronopyranosyl residues [25]. GC/MS analysis of *A. seyal* gum revealed the presence of several phytoconstituents, including 4-methylcatechol; 2,5-diamino-4,6-dihydroxypyrimidine; dihydrouracil; 2-acetyl-3-hydroxy-5,6,8-trimethoxy-1,4-naphthoquinone; fisetin; ferulic acid; resveratrol; β-citronellol; dihydrocarvone; patchoulol; 5,7,3′,4′-tetrahydroxyflavone; chromone, 5-hydroxy-6,7,8-trimethoxy-2,3-dimethyl; α-bisabolol; isolongifolol; genistin; glycitein; quercetin; vanylglycol; quercetin 3-D-galactoside, among others [32].

## 3. Traditional Uses

Unorganized parts (e.g., acacia gum and acacia extracts) and organized parts (e.g., fruits, stem barks, and roots) of acacia trees have been used since ancient times for medical, nutrition, and economic benefits. From the first Egyptian Dynasty (3400 B.C.), gum Arabic (or gum acacia) was used in crafts for the production of ink (mixture of carbon, gum, and water) and also in human and veterinary medicine [25]. Traditionally, African herbalists also used gum acacia to bind pills and stabilize emulsions and in aromatherapy for applying essential oils. The fruits and bark of the acacia tree had also been used by the local people of Sudan to tan leather or as a dye [33]. *A. seyal* (Del.) is a multi-purpose tree that is cultivated for animal fodder, wood, and charcoal in many countries, such as Sudan, Egypt, Somalia Mozambique, and Namibia [34,35]. Presently, gum acacia is used widely in the food and pharmaceutical industries as an important naturally occurring oil-in-water emulsifier. After many years of vacillation, in June 1999, the Codex Alimentarius and the FAO Joint Expert Committee issued the specification for gum acacia [33]. Commercially, it is also used as a film-forming agent in peel-off masks and candies and as emulsifying agents for the production of beverages and flavor concentrates [18,33,36]. Due to the low emulsification properties of *A. seyal* gum, Bi et al. (2017) [37] have incorporated *A. seyal* gum with β-lactoglobulin through Millard reaction to obtain high-quality conjugate.

## 4. Medicinal Uses

Several studies conducted in recent decades revealed that extracts from the bark of *A. seyal* have antibacterial action [25,38,39], antimalarial effect [40], antimycobacterial effect, cyclooxygenase inhibition effect [41], molluscicidal activity [42], and anticancer activities [43,44]. Acacia gum has been established to possess several therapeutic actions, such as hypoglycemic, antidiabetic, antioxidant, immunomodulatory, and cytoprotective antiulcer, and has prebiotic properties [18,25]. Table 2 shows the traditional uses of the different parts of *A. seyal* in different countries for the treatment of various conditions, such as pneumonia, malaria, joint pain, bleeding, rheumatic arthritis, jaundice, chest pain, diarrhea, skin necrosis, bleeding leprosy, dysmenorrhea, eye infection, stomach ulcers, and respiratory tract infection.

## 5. Pharmacological Relevance and Industrial Applications

Gum Arabic from *Acacia* trees, especially from *A. seyal* and *A. senegal*, has a wide range of pharmacological activities and applications in modern and traditional medicine (Table 3).

These activities were reported in their original articles after biological experiments using the total content (not pure individual compounds) of gum Arabic, which contain mainly macro-polysaccharide contents (more than 80% *w*/*w*) and a small amount of protein (1–3.5% *w*/*w*) in addition to traces of other phytoconstituents, e.g., flavonoids, saponins, polyphenolic compounds/tannins and others. The pharmacological activities of gum Arabic are attributed mainly to the polysaccharide contents, but we cannot also neglect the biological activities of other phytoconstituents or at least their synergistic effects, especially we have no confirmed results of the direct relation between specific *Acacia* phytoconstituent and its direct biological activities.

Besides various pharmacological applications of *A. seyal*, it also has diverse applications in the pharmaceutical and food industries. Table 4 summarizes various pharmaceutical and food applications of *A. seyal*.

## 6. Nutritive Value

*A. seyal* gum is composed mainly of a complex polysaccharide that contains a small number of nitrogenous compounds (proteins). Although it has a low nutritive value as an indigestible polysaccharide complex, it has significant nutritional value as a rich source of soluble dietary fibers. Polysaccharide contents of *A. seyal* gum have low caloric contents that are resistant to digestion by intestinal enzymes [73]. However, it can be fermented by colonic microflora to produce short-chain fatty acids, especially butyrate, with high medicinal values [56]. Furthermore, the soluble dietary fiber contents of *A. seyal* gum can retard the absorption of sugars and fats and consequently has antihyperglycemic and antihyperlipidemic activities [72]. Gum Arabic can absorb and retain a reasonable amount of water inside the gastrointestinal tract and, therefore, can aid digestion, improve gastrointestinal movements, treat diarrhea, and soften the hard stool (treats constipation) in addition to its ability to absorb heavy metals and bacterial toxin from the GIT and decreasing their passage into the systemic circulation. The soluble dietary fibers of gum Arabic has a prebiotic effect that can help the growth of probiotic microflora, e.g., *Lactobacillus* and *Bifidobacterium* [99].

## 7. Patent Literature

The following keywords, namely *Vachellia seyal*, *Acacia seyal*, *seyal*, and *Vachellia*, were selected for patent searching using free databases, such as Espacenet (https://worldwide.espacenet.com/patent/search), Patentscope (https://patentscope.wipo.int/search/en/search.jsf), and USPTO (https://patft.uspto.gov/netahtml/PTO/index.html) database). The keywords were entered into all fields/any field section of the databases on 15 November 2021. The number of references obtained by Espacenet (*Vachellia seyal* = 19; *Acacia seyal* = 469; *seyal* = 528; *Vachellia* = 61), Patentscope (*Vachellia seyal* = 28; *Acacia seyal* = 1058; *seyal* = 1162; *Vachellia* = 96), and USPTO (*Vachellia seyal* = 3; *Acacia seyal* = 474; *seyal* = 569; *Vachellia* = 68) were recorded. All the patent references were combined, and duplicate references were removed. The remaining patents/patent applications were segregated according to their patent family, and one patent of each patent family was analyzed because the specification of all members of one patent family remains the same. The patents/patent applications that explicitly/implicitly cover *A. seyal* were analyzed, and others were excluded. The patent summary, along with the applicant’s name, filing date, priority country, legal status, and patent classification, is provided in Table 5.

## 8. Discussion

The traditional uses (Table 2), pharmacological relevance (Table 3), and the applications in food and pharmaceutical industries (Table 4) of gum Arabic, including *A. seyal* gum, make it a substance of high commercial importance. A total of 30 patents/patent applications on *A. seyal* gum belonging to 29 patent families were identified (Table 5). The first patent related to *A. seyal* was published in 1892, wherein the 30th patent application was published on 2 September 2021. The inventions (patents/patent applications) of *A. seyal* can be categorized based on their utility.

Three inventions were related to the prophylaxis/treatment of kidney and bladder affections [107], angiogenesis inhibitors [118], and osteoporosis [120] using *A. seyal* or its equivalents. However, these publications [107,118,120] did not provide the utility of *A. seyal* with experimental evidence for all types of kidney/bladder diseases, cancers, and osteoporosis/bone diseases. This opens an area of research to assess the activity of *A. seyal* for different types of cancers, kidney diseases, and bone diseases.

Five inventions provided pharmaceutical compositions and devices containing *A. seyal* or its equivalents. These include patient compliant solid composition for intra-oral/buccal delivery of insulin [115], sweetener composition with improved palatability [116,117], an oral device to dispense medicament in an oral cavity [122], and dental synbiotic lozenge offering a controlled time-release of the prebiotics, and probiotic organisms [130]. There exist many non-compliant dosage forms and pharmaceutical devices of different drugs. Therefore, new formulations of such dosage forms utilizing *A. seyal* or its equivalents are foreseeable.

*A. seyal* and its equivalents are widely used in the food industry and cosmetics. Accordingly, many inventions of *A. seyal* have been published on these aspects. These include biopolymers of *A. seyal* with improved physicochemical properties [111,112], water-soluble modified gum Arabic [113], tannin-free talha gum food industry [119], coloring fish meat [121], preparation of nutritious chayote bread [123], peanut protein solid beverage [124], protein-fortified frozen dessert [125], chewing gums and candies coated with a confectionary coating containing *A. seyal* [126], a composition useful for coloration of food, beverages, animal feed, cosmetics, or pharmaceutical compositions [127], improved gum Arabic (*A. seyal*) with specified tannin contents for use in beverages or food [128], functional surfactant/emulgent based on *A. seyal* [129], sugar substitute composition [131], plant proteoglycan for cosmetics [132,135], food supplement comprising a mixture of berberine and resveratrol [134], and water-soluble microencapsulated cannabinoid powder for food (beverage, snacks, baked goods), and cosmetics (lotions, makeup) [136].

Two inventions were related to the agricultural applications of *A. seyal*, and its equivalents have been published. These were related to bloat-safe forage crops with altered nutritional value/increased disease resistance [109], and isolated plant gum polynucleotide or synthetic genes that help to improve gum Arabic production [110]. The authors trust that many new agriculture-related inventions on *A. seyal* are possible in the future.

The quality of *A. seyal* and its equivalents is important for consumer safety as it is used in the food and pharmaceutical industry. This problem has been solved by simple inventions that provide a method for determining the quality of gum Arabic using a near-infrared spectrophotometer in a short time [133]. The gum of *A. seyal* encompasses many chemical constituents, which can be isolated. Accordingly, two inventions related to the process for preparing L-rhamnose [108] and recovering arabinose [114] from gum Arabic have been identified.

The publication of only 30 patents/patent applications during the period of 1892–2021 means a small work has been conducted on the inventions related to *A. seyal*. This creates a scope to develop more inventions related to *A. seyal*. The priority patent applications of the identified patents/patent application were filed in different countries (USA = 12; Japan = 5; China = 4; Germany = 2; Europe = 2; United Kingdom = 1; Australia = 1; Israel = 1; Canada = 1; Italy = 1). It is interesting to note that *A. seyal* is called gum Arabic, but no patent application has been filed from any Gulf/Arabic country. Accordingly, we anticipate *A. seyal*-related patent application filings from the Arabic countries.

## 9. Conclusions

The pharmaceutical/medicinal/traditional/cosmetic uses and nutritive values of gum Arabic from both *A. senegal* and *A. seyal* have pronounced the economic or commercial importance of these acacia trees. In Sudan (the main source of GA worldwide), *A. seyal* gum contributed an average of 10% of gum products until 2011. However, due to its availability and economic values, the average contribution percentage of *A. seyal* gum jumped to almost 60% within the last few years. This is the first review that reveals the inventions and patent data of *A. seyal*, which signals that a lot of innovations are possible for *A. seyal* related to the food/pharmaceutical/cosmetic industries and medical field. The authors recommend exploring these opportunities for the benefit of society.

## Figures and Tables

**Table 1:** Analytical data (percentage values) of precepitated fractionsn of *Acacia seyal* gum Arabic compared with its entire substance according to Li et al. 2020 [25].

Fraction	AY60 (Fraction)	AY 80 (Fraction)	AYS (Fraction)	AY (Entire Substance)
Weight percentage (%)	44	39	2.4	100
Average molecular weight	924,900 Da	ND	ND	ND
% moisture content	12.67 ± 0.04	13.59 ± 0.21	ND	14.41 ± 0.11
% Ash content	4.44 ± 0.01	4.51 ± 0.02	ND	3.50 ± 0.02
% total protein content	0.14 ± 0.01	0.13 ± 0.06	0.45 ± 0.02	0.32 ± 0.02
% neutral sugar content	61.24 ± 3.44	63.82 ± 2.76	67.82± 1.62	60.90 ±2.13
% uronic acid content	15.26 ± 0.25	16.17 ± 0.19	1.83 ± 0.07	17.43 ± 0.62
the total molar percentage (mol%) of rhamnose	2.13	2.24	2.28	3.09
mol% of arabinose	43.54	44.80	40.13	47.29
mol% of galactose	39.38	37.22	49.61	33.00
mol% of galacturonic acid	14.95	15.74	1.54	16.62

ND: not determined.

**Table 2:** Traditional uses of *A. seyal* in some African countries.

Country	Use	Part	Ref.
Kenya	Pneumonia	Bark, stem, trunk, twig	[45]
Kenya	Malaria	Roots	[46]
Kenya	Joint pain	Bark, stems, leaves	[47]
Sudan	Bleeding, leprosy	Bark, leaves	[48]
Sudan	Arthritis, rheumatisms, rheumatoid fever	Wood	[49]
Ethiopia	Intestinal parasites	Roots, leaves	[50]
Ethiopia	Chest pain	Roots	[51]
Uganda	Diarrhea, Viral skin necrosis nodules	Roots, bark, leaves	[52]
Djibouti	Dysentery	Bark, roots	[53]
Algeria, Egypt, Morocco	Infected wounds, fever, dysmenorrhea, eye infections, stomach ulcers, rheumatisms	Seed	[54]
Algeria, Egypt, Morocco	Rheumatisms, respiratory tract infection, gastric ulcer	Gum	[55]

**Table 3:** The pharmacological relevance of gum Arabic.

Pharmacological Activity	Possible Mechanism of Action	Refs.
Antiulcerative effect	It provides an antisecretory and cytoprotective effect on GIT.	[56,57]
Wound healing effect	Inhibits periodontic bacterial growth and early deposition of plaque.	[58]
Protective effect on the reproductive system	GA protects the ovary from oxidative stress damage in mice fed with a high-fat diet and increases sperm and semen qualities in the diabetic rat.	[59]
Hepatoprotective effect	GA decreases serum bilirubin level and other liver function markers (ALT, AST) and decreases symptoms of liver damage by restoring the architecture of liver tissue.	[60]
Activity against adenine-induced renal failure	GA mitigates the adenine-induced inflammation and generation of free radicals, resulting in reduced concentrations of plasma urea and creatinine.	[61]
Activity against Hg-induced nephrotoxicity	It prevented Hg-induced degenerative changes of kidney tissues.	[62]
Activity on renal function	It has a significant reduction in blood urea and creatinine concentrations in diabetic nephropathy patients.	[63,64]
Improvement of chronic renal failure	GA can activate colonic bacteria to produce ureases that hydrolyze urea to NH3 and CO2, NH3 excreted in feces through incorporation into bacterial protein. GA increases serum level of butyrate, which prevents the generation of pro-fibrotic cytokine TGF-B1 that contributes to renal fibroblast.	[65]
Activity against doxorubicin induced-cardiotoxicity	It has significant reduction effects on serum creatine kinase and cardiac lipid peroxides.	[66]
Health benefits on the cardiovascular system	GA showed a significant decrease in systolic and diastolic blood pressure. It has a hypocholesterolemic effect, decreasing low-density lipoproteins (LDL) and very-low-density lipoproteins (VLDL).	[67]
Antioxidant activity	GA increases the activity of superoxide dismutase, catalase, and glutathione peroxidase in the liver of diabetic rats by either directly scavenging free radicals or reactive oxygen metabolites or via increasing the synthesis of antioxidant biomolecules.	[59,61,68,69]
Anti-inflammatory effects	GA fibers decreased inflammatory markers and disease severity scores among rheumatoid arthritis patients.	[70]
Supportive treatment of gout	GA reduces in a dose-dependent manner the serum levels of uric acid, urea, creatinine, and erythrocyte sedimentation rate level while increasing the hemoglobin and packed cell volume.	[71]
Effects on fat metabolism and obesity	GA lowers sugar and fat absorption and lowers the caloric density of the diet. It improves the fat utilization in adipose tissues, alternating the expression of mRNA levels of genes involved in lipid metabolism. It has a downregulation effect on 11β-hydroxysteroid dehydrogenase type 1 and increases the viscosity of gastrointestinal contents, thus delaying the evacuation of GIT and contributing to a feeling of satiety. GA influences the gut hormones and enzymes that regulate food intake, satiety, and pancreatic functions. It has metabolic energy dilution, bulking, and satiety effects and aids fermentation to produce short-chain fatty acids and increase GLP-1 and PYY. GA diminishes intestinal SGLT1 expression and activity and glucose-actuated overweight.	[72,73,74,75,76,77,78,79]
Antihypercholesterolimic effect	GA decreases plasma triglyceride, total cholesterol, low-density lipoprotein (LDL), and very-low-density lipoprotein. GA disrupts the enterohepatic circulation of bile acids, leading to increased bile acid excretion.	[75,80,81,82]
Antidiabetic effect	The gel-forming and viscosity of GA inhibit intestinal absorption of macronutrients, enhancement of insulin sensitivity, and modification of certain gut hormones secretion affects a variety of metabolic and inflammatory biomarkers.	[83,84,85,86,87]
Immunomodulatory effects	GA increased the percentage of CD11c+CD40+, CD11c+MHCII+, CD11c+CD86+, and CD54− expressing DCs; in addition, it stimulated the production of IL-6, IL-10, IL12p70, and TNF-α in a p38- and/or extracellular signal-regulated kinases (ERK)-dependent manner.	[59,88]
Antibacterial activity	Due to poly-phenolic (tannins) and saponin contents, GA has antibacterial activities against pathogenic bacteria. GA can also stimulate the growth of probiotic bacteria that protect the body against pathogenic bacteria.	[79,89]
Anti-sickle-cell anemia	GA increases fetal hemoglobin (HbF) level, mean corpuscular volume, and hematocrit level.	[90]
Antimalaria effect	GA metabolites (short-chain fatty acids) increase the level of HbF, which is known to hamper the intra-erythrocytic growth of Plasmodium parasites.	[59,91]
Anticarcinogenic effect	GA modifies cancer-related genes’ mRNA expression. Antioxidant amino acids contents of GA have radical scavenging activities. GA is involved as a nanomaterial for the preparation of anticancer nano-pharmaceuticals, e.g., gold nanoparticles and selenium nanoparticles. GA decreased the colonic mRNA levels of the angiogenetic factors and diminished ss-catenin expression.	[59,69,79,92,93]
Dermatological activity	It is used as an antiallergic, smoothing, protective, binding, and/or stabilizing agent in cosmetic preparations. It has an anti-inflammatory effect against Kwashiorkor skin lesions and decreases skin inflammation (redness).	[94,95,96]
Water and electrolyte up-taking	GA increases water and electrolyte movement from the intestinal lumen to the bloodstream.	[97]
Gut probiotic effect	GA increases the growth of colonic beneficial strains of Lactobacillus and Bifidobacterium. GA selectively nourishes gut microbiota and aid to produces short-chain fatty acids, especially butyrate, and inhibits pathogenic organisms, e.g., the Clostridium histolyticum group, that are commonly associated with gut dysbiosis.	[98,99]
Dentistry applications	It upgrades dental re-mineralization and has some antimicrobial effects. It showed antiplaque on the gums and teeth and anti-gingivitis actions.	[68,100]

**Table 4:** Importance of gum Arabic in food and pharmaceutical industries.

Industrial Relevance	Its Role	Refs.
Adjustment of medication delivery	GA microspheres facilitate absorption and expand the bioavailability of drugs.	[101]
Nanotechnology	GA is a renewable, biocompatible, biodegradable, and non-harmful nanomaterial. GA has the optimum capacity to experience simple synthetic alterations with higher economic values.	[102]
Additive in Food and pharmaceutical industry	GA has many applications as an emulsifier, stabilizer, thickener, processing aid, firming agent, texturizer, adhesive, plasticizer, and formulation aid. GA protects against unstable oils and flavors from the development of rancidity and off-tastes.	[67,103,104,105,106]
Confectionery industry	GA prevents sugar crystallization, modifies texture, emulsifies, acts as a binder, and keeps fatty components evenly distributed.	[103]
Baking products	GA has comparatively low water absorption and favorable adhesive properties. It imparts stability in bun glaze with free-flowing and adhesive characteristics.	[103]
high-quality emulsifying conjugate	*A. seyal* gum was incorporated with β-lactoglobulin through Maillard reaction to obtain emulsifying conjugate with high-quality properties.	[37]

**Table 5:** Summary of the patents/patent applications related to *A. seyal*.

S. No.	Patent/Patent Application Number (Applicant; Publication Date; Priority Country)	International Patent Classification	Status on 15 November 2021 (Family Members)	Summary
1	**US481815A** (Thomas Page; 30 August 1892; USA)	A61K36/48 (EP, US)	Expired patent (None)	It claims a medical composition comprising an aqueous solution (prepared in boiling water) of *Acacia constricta* or its equivalent such as *A. seyal* (two parts) to treat/cure kidney and bladder affections [107].
2	**US5077206A** (Unilever Patent Holdings; 31 December 1991; United Kingdom)	C07H3/08 C12P19/02 C12P19/14 (IPC1-7): C07G17/00 C07H15/00; C12N9/24; C12P19/14;	Expired patent (AT92109T CA1333780C DE3882655T2 EP0317033B1 ES2058241T3 JPH02502248A MX170209B PT89040B WO8904870A1)	It claims an enzymatic process for preparing L-rhamnose from plant material such as *A. Seyal* [108].
3	**WO9807836A1** (Commonwealth Scientific and Industrial Research; 26 February 1998; Australia)	C07K16/40, C12N15/29, C12N15/82, C12N9/04, (IPC1-7): A01H1/00, C12N15/29, C12N15/53, C12N15/61, C12N9/02, C12N9/90	Lapsed (AR009294A1, CA2264201A1, NZ334224A)	It claims isolated nucleic acid molecules that encode leucoanthocyanidin reductases of plants such as *A. seyal* [109].
4	**US6570062B1** (Ohio University; 27 May 2003; USA)	C07K14/415, C12N15/29, C12N15/82, (IPC1-7): C12N15/29, C12N15/82, C12P19/04, C12P21/02	Expired patent (WO9903978A1)	It claims an isolated plant gum polynucleotide or synthetic genes that help to improve gum Arabic production in plants (*A. senegal* and *A. seyal*) [110].
5	**US6610810B2** (Phillips Hydrocolloids Research Limited; 26 August 2003; USA)	A61L27/00, C08B11/12, C08B11/20, C08B37/00, C08B37/06, C08F2/46, C08G63/00, C08H1/06, C08H6/00, C08H7/00, C08J3/28 (IPC1-7): C08F2/46, C08G63/00, C08H5/02	Expired patents (CA2440863A1 EP1565483A2 JP2004536624A RU2280038C2 WO02072862A2 ZA200307398B)	**US6610810B2** [111] and **US6841644B2** [112] are members of the same patent family and claim new biopolymers of *A. seyal* with improved physicochemical properties.
6	**US6841644B2** (Phillips Hydrocolloids Research Limited; 11 January 2005; USA)			
7	**WO2004089992A1** (Phillips Hydrocolloids Research Limited; 21 October 2004; Japan)	A23L1/308, A23L29/20, A61K31/736, A61P1/14, A61P3/06, A61P3/10, A61P35/00, C08B37/00 (IPC1-7): A23L1/308, A61K31/736, A61P1/14, A61P3/06, A61P3/10, A61P35/00, C08B37/00	Lapsed (CA2521692A1, CN100447160C, EP1612225A1, JPWO2004089992A1, US2006240166A1)	It claims a water-soluble modified gum Arabic that has a total dietary fiber content of 90% or more, which was prepared by heating gum Arabic (*A. seyal* and *A. senegal*) [113].
8	**WO2005042788A1** (Danisco Sweeteners Oy; 12 May 2005; USA)	C07H1/08, C13K13/00, (IPC1-7): C13K13/00	Lapsed (EP1678330A1, NO20062457L, US2005096464A1, US2007112187A1)	It claims a process of recovering arabinose from vegetable fiber (exudate gum such as gum Arabic, gum ghatti, and gum tragacanth) [114].
9	**WO2006103657A2** (Dexcel Pharma Technologies; 5 October 2006; Israel)	A61K9/006 (EP), A61K9/127 (EP), A61K9/7007 (EP)	No national phase entry (None)	It claims a solid composition for intra-oral/buccal delivery of insulin encompassing insulin, a hydrophilic polymer (such as gum Talha or *A. seyal*) matrix, and a phospholipid (such as lecithin or phosphotidylcholin), providing insulin bioavailability of 5–20% [115].
10	**US9011956B2** (Prakash Indra; 21 April 2015; USA)	A23L27/00, A23L27/30, A23L1/305, A23L2/52, A23L2/60, A61K31/575	Patented case (AR056220A1, AU2006318781B2, BRPI0618945A2, CA2630049C, CA2969364C, CN103393062B, DK2526783T3, EP2526778B1, EP2526783B1, EP3199033B1, ES2611887T3, JP6113974B2, JP6609587B2, KR101374346B1, KR101385710B1, MX2008006583A, MY149619A, TW200738168A, UY29928A1, WO2007061795A1, ZA200804458B)	It claims a sweetener composition comprising rebaudioside A (purity > 97%), erythritol, and a sweet taste improving polymer (such as gum *A. senegal* and gum *A. seyal*) or combinations thereof [116].
11	**US2007275147A1** (The Coca-Cola Company; 29 November 2007; USA)	A23L27/00, A23L27/30	Abandoned in 2011 (AR056180A1, AU2006335251A1, BRPI0619068A2, CA2629556A1, EP1965667A2, JP2009517037A, KR20080071606A, MX2008006587A, TW200738169A, WO2007081442A2)	It claims a synthetic sweetener composition with an improved taste profile comprising a sweet taste improving polymer (*A. senegal* and gum *A. seyal*) [117].
12	**WO2008074437A2** (Eberhard-Karls-Universitaet Tuebingen Universitaetsklinikum; 26 June 2008; Germany)	A61K35/00, A61K36/48	Lapsed (DE102006061517A1, EP2109453A2)	It claims the use of gum Arabic (*A. senegal* and *A. seyal*) as an active ingredient of an angioinhibin (angiogenesis inhibitors) [118].
13	**JP5139719B2** (Kamisu Kagaku; 6 February 2013; Japan)	C02F1/58, C08B37/00	Patented case (None)	It claims a tannin-free talha gum (*A. seyal*) having acceptable quality for use in the food industry [119].
14	**WO2009021661A1** (Eberhard-Karls-Universitaet Tuebingen Universitaetsklinikum; 19 February 2009; Germany)	A61K36/48, A61P19/10	Lapsed (DE102007039310A1)	It claims the use of gum Arabic (*A. senegal* and *A. seyal*) for the prophylaxis and treatment of osteoporosis [120].
15	**CN102845737A** (Tianjin Tiankangyuan Biological Technology; 2 January 2013; China)	A23L1/275, A23L17/00	Withdrawn (None)	It claims a method for uniformly coloring fish meat (salmon meat) utilizing a 10% aqueous solution of *A. senegal* or *A. seyal* [121].
16	**US20130177867A1** (Morales Anthony; 11 July 2013; USA)	A61C19/06, A61C5/14, A61J17/00	Abandoned in 2014 (None)	It claims an oral device to dispense substances in an oral cavity comprising a natural gum (gum Arabic such as *A. senegal* and *A. seyal*) or a combination of natural gum with a medicament [122].
17	**CN105341064A** (Li Hua; 24 February 2016; China)	A21D13/00, A21D2/36	Withdrawn (None)	It claims a nutritional chayote bread containing gum Arabic (*A. senegal* and *A. seyal*) [123].
18	**CN105341611B** (Chinese Academy of Agricultural Sciences; 29 June 2018; China)	A23L2/39, A23L2/62	Patented case (None)	It claims a stable and nutritional peanut protein solid beverage comprising gum Arabic (*A. senegal* and *A. seyal*) [124].
19	**US2017071229A1** (Coolwhey Inc.; 16 March 2017; USA)	A23G9/32, A23G9/38	Abandoned in 2018 (CA2942266C)	It claims a protein-fortified frozen dessert formulation utilizing gum Arabic (*A. senegal* and *A. seyal*) as a stabilizing agent [125].
20	**US20130101706A1** (Haseleu Andrea; 25 April 2013; USA)	A23G3/38, A23G3/54	Abandoned in 2017 (AU2010336955B2 CA2785060C CN102695425A EP2519113A1 EP2519113B1 PL2519113T3 RU2517862C2 WO2011082050A1)	It claims a confectionary product (chewing gums and candies) comprising a film-forming agent (gum tahla) [126].
21	**US8680161B2** (Hitzfeld Andrea; 25 March 2014; Europe)	A23L33/155, A61K31/07	Patented case (CN102056497B, EP2280611B1, ES2436167T3, JP2011521658A, PL2280611T3, WO2009147158A2)	It claims a composition of dried particles of gum ghatti, gum *Acacia* (*A. senegal* and *A. seyal*), and at least one fat-soluble active ingredient (carotenoid) useful for the enrichment, fortification, and/or coloration of food, beverages, animal feed, cosmetics, or pharmaceutical compositions [127].
22	**EP3328901B1** (Döhler GMBH; 11 September 2019; Europe)	A23L29/25, C08B37/00, C08L5/00, A23L2/52	Patented case (BR112018001226A2, ES2758363T3, HRP20192112T1, HUE046765T2, JP2018523494A, MX2018001147A, PL3328901T3, PT3328901T, RS59685B1, RU2725959C2, SI3328901T1, US2018215841A1, US2021070892A1, WO2017017248A1)	It claims an improved gum Arabic (*A. seyal*) having a tannin content > 700 ppm (w/w) with superior emulsification performance [128].
23	**CN106377450A** (Chen Xiong; 8 February 2017; China)	A23L29/10, A23L33/10, A61K36/14, A61K47/36, A61K8/73, A61K8/9761, A61P11/10, A61P11/14, A61P9/12, A61Q5/02, A61Q5/12	Withdrawn (None)	It claims a functional surfactant/emulgent based on *A. seyal* gum [129].
24	**US20170232048A1** (Renuzoral; 17 August 2017; USA)	A61K31/715, A61K31/723, A61K31/733, A61K35/744, A61K35/747, A61K36/48, A61K47/02, A61K47/10, A61K47/12, A61K47/26, A61K47/36, A61K47/38, A61K47/46, A61K8/02, A61K8/24, A61K8/34, A61K8/36, A61K8/60, A61K8/73, A61K8/97, A61K8/99, A61K9/00, A61Q11/00	Abandoned in 2019 (None)	It claims a dental synbiotic lozenge encompassing adhesive prebiotics (inulin, *A. seyal* gum, Konjac mannan, Xanthan gum) and one or more species of probiotic organisms [130].
25	**US2020187535A1** (FTC International Consulting Ltd.; 18 June 2020; Canada)	A23L27/00, A23L27/30, A23L33/21	Under examination (CA3063233A1 MX2019013378A WO2018205039A1)	It claims a sugar substitute composition comprising a digestion resistant soluble fiber (*A. senegal* and *A. seyal*) between 99.00% and 99.99% by weight and a stevia leaf extract (0.01% and 1.00% by weight) [131].
26	**JP2019172718A** (NOF Corporation; 10 October 2019; Japan)	A61K36/48, A61K38/02, A61K8/73, A61K8/9789, A61P17/16, A61P43/00, A61Q19/00, C08B37/00	Under examination (None)	It claims a plant proteoglycan (molecular weight of 900,000-3,500,000; total aldehyde content = 2.0 μmol equivalent/g or less) obtained from *A. senegal* or *A. seyal* [132].
27	**JP2019191085A** (Sanei Gen FFI Inc.; 31 October 2019; Japan)	G01N21/359	Under examination (None)	It claims a method for determining the mixing/contamination of different types of gum into gum Arabic (*A. senegal* and *A. seyal*) or gadhi gum by measuring diffuse reflection using a near-infrared spectrophotometer [133].
28	**WO2020128802A1** (S&R Farmaceutici; 25 June 2020; Italy)	A61K31/05, A61K31/4375, A61P3/06	Entered into national phase (CA3122918A1 CN113242733A EP3897596A1 IT201800011155A1)	It claims a food supplement comprising a mixture of berberine, resveratrol, and one nutrient with properties of regulating the lipid profile (*A. senegal* and *A. seyal*) for use in the treatment and/or control of dyslipidemia [134].
29	**WO2021059344A1** (NOF Corporation; 1 April 2021; Japan)	A61K8/9789, A61Q19/00, C08B37/00, A23L33/105, A61K38/02	No national phase entry (None)	**WO2021059344A1** [135] claims the same invention as described in **JP2019172718A** [132].
30	**US20210267907A1** (Prinova Flavors; 2 September 2021; USA)	A61K31/05, A61K9/50	Under examination (CN113304703A, EP3875076A1)	It claims a water-dispersible microencapsulated composition containing 10–20% of cannabinoid and at least one gum *Acacia* (*A. senegal* and *A. seyal*) for use as an ingredient in food and cosmetics [136].

## Data Availability

This study did not report any data.
